# Higher hospital volume reduces early failure rates in single‐stage revision TKR for infection: An analysis of the United Kingdom National Joint Registry and National Administrative Databases

**DOI:** 10.1002/ksa.12578

**Published:** 2025-01-15

**Authors:** Alexander H. Matthews, William K. Gray, Jonathan P. Evans, Ruth Knight, Jonathan T. Evans, Sarah E. Lamb, Tim Briggs, Andrew Porteous, Shiraz A. Sabah, Abtin Alvand, Andrew Price, Andrew D. Toms

**Affiliations:** ^1^ Getting It Right First Time Programme, NHS England London UK; ^2^ Royal Devon University Healthcare NHS Foundation Trust Exeter UK; ^3^ University of Exeter Exeter UK; ^4^ Nuffield Department of Orthopaedics, Rheumatology, and Musculoskeletal Sciences University of Oxford Oxford UK; ^5^ Liverpool Clinical Trials Centre University of Liverpool Liverpool UK; ^6^ University of Exeter College of Life and Environmental Studies: University of Exeter Faculty of Health and Life Sciences Exeter UK; ^7^ Royal National Orthopaedic Hospital London UK; ^8^ North Bristol NHS Trust Bristol UK; ^9^ Nuffield Orthopaedic Centre Oxford UK

**Keywords:** complications, infection, mortality rates, revision rates, revision total knee replacement, volume‐outcomes

## Abstract

**Purpose:**

Revision knee replacement (RevKR) for infection is rare but increasing. It is hypothesised that higher hospital volume reduces adverse outcomes. The aim was to estimate the association of surgical unit volume with outcomes following first, single‐stage RevKR for infection.

**Methods:**

This population‐based cohort study merged data from the United Kingdom National Joint Registry, Hospital Episode Statistics, National Patient Reported Outcome Measures and the Civil Registrations of Death. Patients undergoing procedures between 1 January 2009 and 30 June 2019 were included. Early outcomes were chosen to reflect the quality of the surgical provision and included re‐revision at 2 years, mortality, serious medical complications, length of stay and patient‐reported outcome measures (PROMs). Adjusted fixed effect multivariable regression models were used to examine the association between surgical unit mean annual caseload and the risk of adverse outcomes.

**Results:**

A total of 1477 patients underwent first‐time single‐stage RevKRs for infection across 267 surgical units and 716 surgeons. Following adjustment for age, gender, American Society of Anaesthesiologists grade, surgeon volume, year of surgery and operation funder and modelling surgical unit volume with restricted cubic spline, a greater mean annual volume was associated with a lower risk of re‐revision at 2 years. The odds of re‐revision in hospitals performing fewer than or equal to 12 cases per year was 2.53 (95% confidence interval = 1.50–4.31) times more likely than hospitals performing three to four cases per month. Annual variation in surgical unit volume was not associated with mortality and serious medical complications within 90 days. Only 99 out of 1477 (7%) of patients had linked PROMs which precluded subsequent analysis.

**Conclusion:**

Overall, higher volume surgical units had lower rates of early re‐revision following the first RevKR for infection. We were unable to provide recommended specific volume thresholds for units; however, the probability of re‐revision appears to be lowest in the highest volume units.

**Level of Evidence:**

Level III, retrospective cohort study of prospectively collected data.

**Abbreviations**
ASA: American Society of Anaesthesiologists
CI: confidence interval
EQ‐5D: EuroQol 5 Dimension
HES: APC Hospital Episode Statistics Admitted Patient Care
MDT: multidisciplinary team
NHS: National Health Service
NJR: National Joint Registry
OKS: Oxford Knee Score
OR: odds ratio
PROM: patient‐reported outcome measure
pTKR: primary total knee replacement
RevKR: revision knee replacement
TKR: total knee replacement

## INTRODUCTION

Revision knee replacement (RevKR) is a complex and expensive orthopaedic procedure associated with high rates of further surgery and mortality [27]. About 19.9% of first RevKRs are re‐revised within 13 years [8]. The incidence of RevKR has increased steadily over the past 15 years in the United Kingdom [26], despite improvements in clinical outcomes following primary total knee replacement (pTKR) [11]. This is due to increases in the utilisation of joint replacement, especially in younger patients and greater life expectancy [22]. The demand for primary TKR in patients aged <65 years is increasing at a faster rate than in older patients [17]. Younger patients are also associated with the highest overall re‐revision rates [8], and some projections suggest that patients <65 years will soon comprise over 50% of the RevKR burden [16].

RevKR services in England have recently undergone major service reconfiguration to form regional revision networks [15]. The framework underpinning these networks is based on major trauma and cancer services where more complex work is triaged to larger specialist centres [12, 23]. A revision network consists of a high‐volume centre with established clinical experience and access to other relevant services such as vascular surgery, plastic surgery and specialist orthopaedic microbiology [34]. Such centres become major revision centres and coordinate the regional network performing the more complex case mix. Most hospitals performing RevKR will be considered revision units, and some very low‐volume centres will become primary arthroplasty units and stop routinely performing RevKR [15].

An important assumption of revision networks is that a positive patient volume–outcome relationship exists; that is, higher volume surgical units achieve better clinical outcomes. This relationship has previously been identified in primary hip, knee and shoulder replacement surgery [18, 33, 35]. Evidence for RevKR is more limited with an emerging body of evidence from smaller studies focusing on the incidence of early re‐revision surgery, being broadly [13, 25, 37], but not uniformly [36], supportive of a positive surgical unit volume–outcome relationship. There is also significant bias due to confounding when comparing the results between these studies. Four registry‐based studies have been published in the literature to date [19, 20, 36, 37], with only one study adjusting for age, gender, comorbidity, indication for revision and surgeon volume in the analysis of outcomes [37].

Therefore, the aim of the present study was to investigate the relationship between surgical unit caseload and patient‐relevant outcomes following first, single‐stage RevKR for infection. Infection represents a more complex type of RevKR which carries the highest re‐revision rates [31] and represents the most common indication for RevKR [26]. We defined patient‐relevant outcomes related to hospital volume as re‐revision within 2 years of the index procedure; mortality and serious medical complications within 90 days and knee function at 6 months following revision surgery. The null hypothesis was that the surgical unit caseload was not associated with the primary or secondary outcomes. The alternative hypotheses were that a higher surgical unit caseload was associated with a lower re‐revision rate at 2 years, a lower mortality rate at 90 days, a lower rate of any serious medical complication at 90 days, improved knee function and quality of life and reduced length of stay.

## METHODS

### Study design

**Figure 1:**
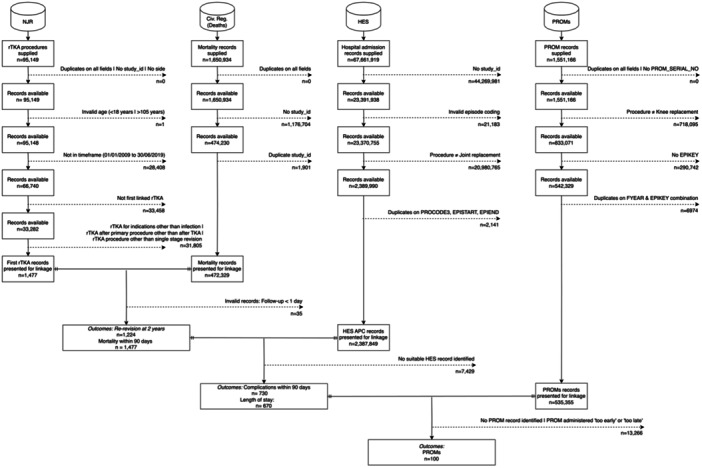
Flowchart demonstrating the attrition of study records during data preparation and record linkage.

In this retrospective observational registry‐based study, we analysed data from the National Joint Registry (NJR), which covers procedures performed at public and private hospitals in England, Wales, Northern Ireland, the Isle of Man and the States of Guernsey. This is the largest NJR in the world. These data were linked with other routine health data, including Hospital Episode Statistics Admitted Patient Care (HES APC); National Health Service (NHS) patient‐reported outcome measures (PROMs) and the Civil Registrations of Death registers. Data cleaning and preparation of the NJR, HES APC data, Civil Registrations of Death and NHS PROMs followed a previously published and reproducible workflow [31]. Data linkage and attrition are demonstrated in Figure 1.

### Participants

All first‐time, single‐stage RevKR procedures for infection performed between 1 January 2009 and 31 December 2017 were included. This timeframe was selected (i) to provide a minimum 2‐year follow‐up period to 31 December 2019 (which preceded the Covid‐19 pandemic, where access to RevKR surgery was limited and unlikely to be representative of usual care [10]), and (ii) to exclude early NJR records where compliance with the registry was low [30]. RevKR was defined as ‘(any) operation performed to remove (and usually replace) one or more components of a total joint prosthesis for whatever reason’ [24]. Therefore, revisions following partial knee replacement and procedures that could not be linked to a pTKR were excluded from the analysis of unit‐level outcomes. However, these revision procedures did contribute to caseload calculations. *First*, RevKR was defined as the earliest revision procedure following a pTKR recorded on the NJR. Indication for revision was categorised hierarchically into 10 discrete groups: Infection, Malalignment, Loosening/Lysis, Component wear, Instability, Fracture, Progressive Arthritis, Stiffness, Unexplained Pain and Others. Where multiple reasons for revision were recorded, a hierarchy of revision was used to determine the primary reason for revision and the revision was attributed to the highest‐ranking reason. This was based on the Australian Orthopaedic Association National Joint Replacement Registry hierarchical system [3]. We included only procedures where the primary reason for revision was infection. All procedures meeting any of the following criteria were excluded: intention to treat in multiple stages; amputation, arthrodesis procedures; and missing, out‐of‐range or invalid entries in key date fields or other fields necessary for case‐mix adjustment. Multiple‐stage procedures were excluded because these would automatically trigger a planned repeat operation within 365 days of the index revision, which would bias our primary outcome measure. Ethical approval was obtained from the London‐Bromley Research Ethics Committee (20/LO/0428). Data access approvals were obtained from the NJR for England, Wales, Northern Ireland, the Isle of Man and the States of Guernsey (NJR) (RSC2017/26). Patients who did not consent to the NJR audit were not included.

### Variables

The exposure of interest was the caseload of each surgical unit. Annual *caseload* was defined as the number of RevKR performed by a surgical unit in the 365 days prior to each index procedure. Caseloads were calculated before applying inclusion and exclusion criteria (except for duplicates and unreliable date information) to avoid endogeneity. This meant that RevKR for all indications, re‐revision procedures, revisions of partial knee arthroplasties and RevKR not linked to a pTKR were all included in the estimate of caseload. Each stage of a staged procedure was counted separately. For RevKR procedures in 2009, caseloads were calculated using data from 2008 if appropriate. For surgical units newly formed over the study period, RevKR procedures within the first 365 days contributed to caseload calculations but not to outcome assessment. The earliest recorded revision procedure in each surgical unit was obtained using an extended NJR data set.

For modelling, surgical unit caseloads were converted into mean annual volumes, as described below [35]. The following four groups of known or potential confounding variables were considered in our multivariable logistic regression modelling in the following order:

*Patient factors*: Age in years (continuous), gender (male/female) and American Society of Anesthesiologists (ASA) grade (1, 2 and 3+) were obtained from the NJR. Pre‐operative PROMS (Oxford Knee Score [OKS] and EQ‐5D) were found in the linked National PROMs.

*Surgical factor*: Mean surgeon volume (continuous) derived in the same way as surgical unit volume was calculated. This was the average of the number of RevKR performed by a consultant in the 365 days prior to each index procedure across the entire cohort.

*Hospital factor*: The NJR collects information on hip, knee, ankle, elbow and shoulder joint replacement surgery performed at both public and private hospitals. Data were collected on whether the operation was funded by NHS providers or independent sector providers. Anecdotal evidence suggests simpler RevKR may be performed in independent surgical sites where access to specialist services such as intensive care facilities is limited.

*Temporal factor*: Calendar year of procedure (2009, 2010, 2011, 2012, 2013, 2014, 2015, 2016, 2017, 2018 and 2019).

### Outcome measures

The primary outcome was the rate of re‐revision at 2 years. A 2‐year timeframe was selected under the hypothesis, agreed by a consensus of experts in the field, that re‐revisions within this *early* period may be more representative of the quality of surgical provision rather than *late* revisions (which might, e.g., be due to expected component wear or loosening over time, or infection from a secondary source) [21, 34]. This early period also correlates with that reported in the existing literature [31].

Secondary outcomes were:

*90‐day all‐cause mortality*, identified using linked data from Civil Registrations (Mortality) data set.

*Rate of any serious medical complication within 90 days*, identified using the HES APC data set following the previously described methodology [31].

*Post‐operative knee function*, identified using the OKS [28] at 6 months following RevKR from the NHS PROMs data set. The OKS is a 12‐point Likert item questionnaire that has been shown to have good measurement properties for the evaluation of knee function following RevKR [28]. The worst sum score is 0 points, and the best sum score is 48 points.

*Post‐operative health‐related quality of life using the EuroQol 5 Dimension (EQ‐5D) index* [9] *at 6 months following RevKR from the NHS PROMs data set*. The EQ‐5D analyses five dimensions (mobility, self‐care, usual activities, pain/discomfort and anxiety/depression), each rated from 1 (*no problems*) to 3 (*severe problems*) [9]. The EQ‐5D profile is converted to a utility for the UK population, where best health is represented by 1 and worst health is represented by −0.59. A health state of 0 is equivalent to death.

*Inpatient length of hospital admission* identified using the HES APC data set attributed to continuous inpatient spells, which is the preferred estimate of length of stay [7]. This refers to the length of the first stay after the operation, regardless of any transfers across providers. The median length of stay was calculated after visually inspecting the distribution, and this was dichotomised into prolonged length of stay if longer than the median stay.

### Statistical analyses

*Mean annual volume* was defined as the mean of the caseload across all candidate procedures for a surgical unit. This was calculated using group mean centring [35]. The distribution of this new variable was inspected using histograms and empirical cumulative frequency distributions. Continuous variables were described using means and standard deviations or medians and interquartile ranges as appropriate, after visual inspection of data distributions. Categorical data were described using counts with percentages. For descriptive analyses, mean surgical unit caseloads were categorised into four groups (less than 12 cases per year, which corresponds to 1 RevKR per month; 13–24 cases per year, corresponding to 1–2 RevKR per month; 25–52 cases per year, corresponding to 3–4 RevKR per month; 53 or more cases per year, corresponding to more than 1 RevKR per week). These volume groups were based on the classification described by Halder et al. [13].

During the modelling of outcomes surgeon volume, surgical unit volume and age were treated as continuous variables. The relationship between mean surgical unit volume and the primary outcome was found to be non‐linear. Mean surgical unit volume was modelled with restricted cubic splines to allow for the non‐linear effects when testing the association with the primary outcome only. The Akaike information criterion (AIC) was used to select the most parsimonious specification of restricted cubic splines using the final adjusted model. Despite its non‐linear relationship with the primary outcome, age was modelled as a linear term as modelling with splines did not improve the model fit and none of the spline terms were statistically significant. Surgeon volume was not found to be non‐linear with the outcome.

Fixed effects logistic regression models were used for the outcomes of re‐revisions, serious adverse events (dichotomised into the presence of any medical complications or none), prolonged length of stay and mortality at 90 days. Fixed effects linear regression models were used for post‐operative PROMs. Adjustment for confounding was undertaken incrementally, adjusting for each of the four groups of confounding variables to explore their influence on the volume effect at each stage with reference to model fit statistics. This was done following an a priori methodology with the addition and or removal of factors in the following order: patient factors, surgical factors, hospital factors and finally temporal factors. The ultimate decision on the preferred statistical model was assessed using the AIC accepting the model with the lowest AIC. Multicollinearity was assessed using eigenvalues, variance inflation factors and by examination of model parameter estimates with stepwise addition and removal of covariates. Odds ratios (ORs) with 95% confidence intervals (CIs) and associated *p* values were reported. A *p *< 0.05 was taken to indicate statistical significance. Inspection of residuals and fitted values, *Q*–*Q* plots and Cook's distance plots were assessed to ensure assumptions of linear regression were satisfied. Data analysis was performed using R version 4.3.1.

## RESULTS

**Figure 2:**
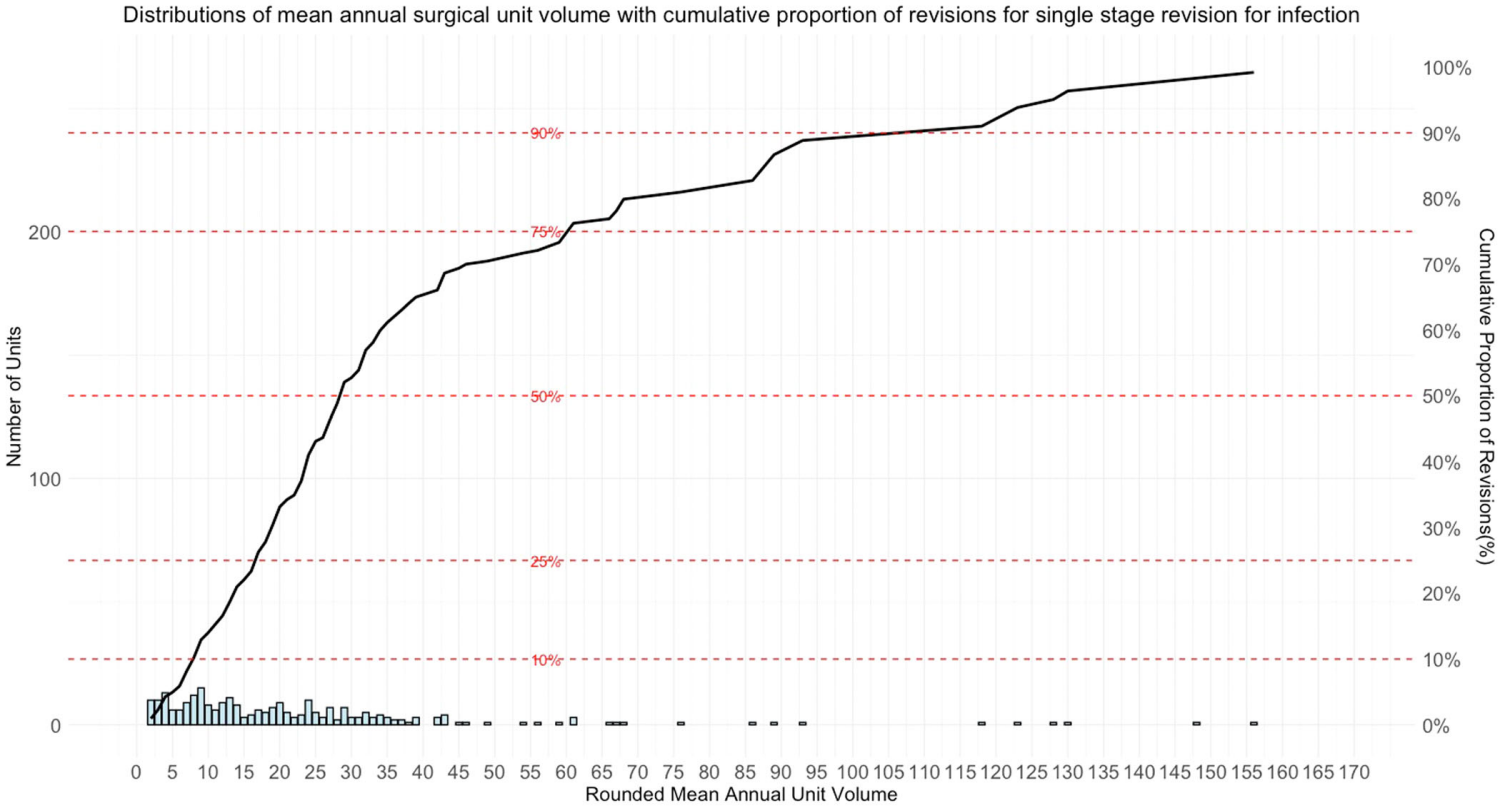
Histogram showing the distribution of surgical unit mean annual revision knee replacement (RevKR) volumes of all revisions for only surgical units who undertook single‐stage RevKR for infection between 1 January 2009 and 30 June 2019. Surgical unit mean annual volumes were grouped by rounding down to the nearest integer. The black line indicates the cumulative proportion of total RevKR cases performed by surgical units at or below each indicated annual volume group. Horizontal dashed lines indicate the 10th, 25th, 50th, 75th and 90th percentiles.

**Figure 3:**
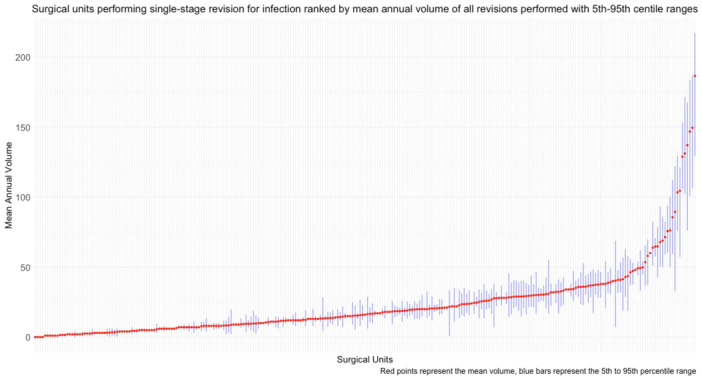
Caterpillar plot showing mean (red points) and 95th and 5th percentiles (blue shaded lines) of annual revision knee replacement (RevKR) volumes for all revisions for each of the 267 surgical units who undertook single‐stage RevKR cases for infection between 1 January 2009 and 30 June 2019.

A total of 71,285 RevKR were recorded in the NJR between 1 January 2008 and 30 June 2019, as shown in Figure 1. This included revisions for all indications of revision and those with previous RevKRs. After applying the eligibility criteria, 33,282 first‐time RevKR were recorded between 1 January 2009 and 30 June 2019 for all causes. These included 1477 single‐stage revisions for infection. These procedures were performed on 1477 patients in 267 surgical units by 716 surgeons. Descriptive statistics summarising co‐confounding variables by mean annual surgical unit volume are shown in Table 1. A higher proportion of surgical units are performing lower mean annual volumes of single‐stage revisions for infection, as shown in Figure 2. Half of the total revisions performed were done by units with a mean annual volume of 28 or less. The highest volume surgical units had greater variability in annual operating volumes, as shown in Figure 3. Baseline demographics, including age, gender, ASA grade and operation funder, were broadly similar across volume categories (see Table 1). Differences were observed across volume categories for median surgeon volume; however, there was an overlap of interquartile ranges across all volumes. The number of procedures available for the analysis of re‐revision, serious adverse events, mortality and length of stay in the linked NJR and Hospital Episode Statistics ranged from 1076 to 1319. Only 99 cases had completely linked preoperative and post‐operative PROMs data and this precluded the analyses of PROMs in the secondary outcomes.

**Table 1:** Descriptive statistics by mean annual surgical unit volume, categorised by a priori categorical variables.

	Mean annual surgical unit volume classification			
	Low	Medium	Medium	High
	≤12	13–24	25–52	≥53
Revisions				
Number of revisions	265 (18.09%)	347 (23.69%)	429 (29.28%)	424 (28.94%)
Surgical units and surgeons				
Number of units	110 (42.15%)	70 (26.82%)	62 (23.75%)	19 (7.28%)
Number of surgeons	185	204	235	152
Median annual surgeon volume	5.99 (3.11–9.71)	6.95 (3.89–11.95)	9.08 (4.40–14.33)	17.96 (8.97–29.88)
Age				
Mean age (SD)	70.25 (10.40)	69.35 (9.65)	70.59 (9.55)	70.96 (0.23)
60 or less years	48 (18.11%)	55 (15.85%)	58 (13.52%)	65 (15.33%)
61–64 years	28 (10.57%)	50 (14.41%)	47 (10.96%)	45 (10.61%)
65–69 years	51 (19.25%)	69 (19.88%)	87 (20.28%)	80 (18.87%)
70–74 years	36 (13.58%)	73(21.04%)	79 (18.41%)	72 (16.98%)
75–79 years	46 (17.36%)	41 (11.82%)	82 (19.11%)	57 (13.44%)
80 years or more	56 (21.13%)	59 (17.00%)	76 (17.72%)	105 (24.76%)
Gender				
Female	101 (38.11%)	158 (45.53%)	192 (44.76%)	186 (43.87%)
ASA grade				
ASA1	14 (5.28%)	14 (4.03%)	17 (3.96%)	18 (4.25%)
ASA2	151 (56.98%)	194 (55.91%)	245 (57.11%)	248 (58.49%)
ASA3+	100 (37.74%)	139 (40.06%)	167 (38.93%)	158 (37.26%)
Year of surgery				
2009	11 (4.15%)	10 (2.88%)	19 (4.43%)	10 (2.36%)
2010	15 (5.66%)	17 (4.90%)	17 (3.96%)	17 (4.01%)
2011	10 (3.77%)	20 (5.76%)	17 (3.96%)	19 (4.48%)
2012	22 (8.30%)	26 (7.49%)	26 (6.06%)	25 (5.90%)
2013	15 (5.66%)	25 (7.20%)	32 (7.46%)	26 (6.13%)
2014	23 (8.68%)	48 (13.83%)	40 (9.32%)	46 (10.85%)
2015	38 (14.34%)	43 (12.39%)	48 (11.19%)	62 (14.62%)
2016	41 (15.47%)	50 (14.41%)	73 (17.02%)	69 (16.27%)
2017	51 (19.25%)	48 (13.83%)	80 (18.65%)	74 (17.45%)
2018	30 (11.32%)	47 (13.54%)	63 (14.69%)	61 (14.39%)
2019	9 (3.40%)	13 (3.75%)	14 (3.26%)	15 (3.54%)
NHS or independent funding				
NHS	211 (79.62%)	339 (97.69%)	420 (97.90%)	422 (99.53%)
Independent	54 (20.38%)	8 (2.31%)	9 (2.10%)	2 (0.47%)

*Note*: Patient‐level descriptive statistics are according to the mean annual unit volume category. New surgical units without previous 365‐day volume data were excluded from the outcome analysis (261 surgical units and 708 surgeons). Some surgeons appear in multiple surgical unit volume groups and are represented as counts only.

Abbreviations: ASA, American Society of Anesthesiologists; NHS, National Health Service; SD, standard deviation.

### Outcome data

**Figure 4:**
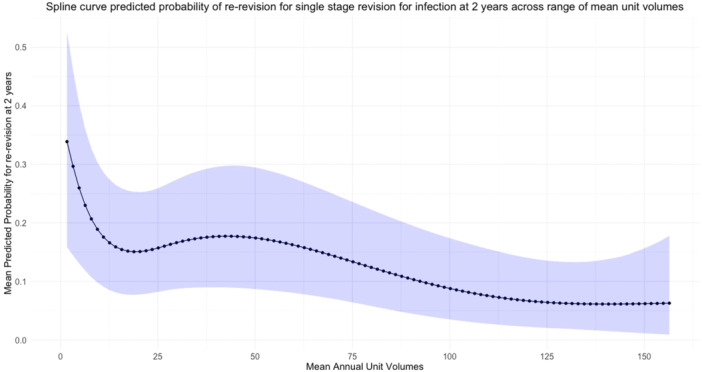
Predicted probability of re‐revision at 2 years by mean unit volume for single‐stage revision knee replacement for infection. A fixed effects multivariable logistic regression model using three knots at 5%, 50% and 95% percentiles of mean unit volume. 95% confidence intervals are represented by blue shaded line.

The crude re‐revision rate at 2 years for first‐time single‐stage revisions for infection across the cohort was 236 out of 1224 (19.3%). The unadjusted rate of re‐revision between volume categories is summarised in Table 1. Following modelling and adjustment of co‐variates, the predicted rate of re‐revision also decreased overall with increasing mean annual surgical unit volume, as seen in Figure 4. Categorising surgical unit volume before modelling demonstrated significant differences in re‐revision favouring higher‐volume centres. The odds of re‐revision in hospitals performing fewer than or equal to 12 cases per year were 2.53 (1.50–4.31) times more likely than the highest volume hospitals (Table 2). We did not observe a plateau in the predicted risk of re‐revision as this continued to reduce with volume. There was not a diagnostically robust change point analysis to determine a minimum volume threshold.

**Table 2:** Multivariable logistic regression modelling of re‐revision at 2 years with mean surgical unit volume in categories.

	Mean annual surgical unit volume classification			
	1–12	13–24	25–52	>52
Odds ratio	Reference	0.5 (0.3–0.8)	0.7 (0.4–1.1)	0.4 (0.2–0.7)
*p*‐value	‐	<0.05	0.1	<0.05

The results for the secondary outcomes are summarised in Table 3. The crude rate of medical complications within 90 days was 87 out of 730 (11.9%). The crude mortality rate within 90 days was 23 out of 1477 (1.6%). There was no statistical association between mean annual surgical unit volume and risk of medical complications (OR 1.0, 95% CI 1.0–1.0) and mortality within 90 days (OR = 1.0 95% CI = 1.0–1.0, *p* = 0.1). Increasing mean annual unit volume was not associated with inpatient hospital stays more than the median of 12 days (OR = 1.0, 95% CI = 1.0–1.0, *p* = 0.6).

**Table 3:** Multivariable regression modelling of secondary outcomes with mean surgical unit volume.

	Association between mean surgical unit volume (continuous) and secondary outcomes		
	Odds ratio/coefficient estimate (95% confidence intervals)	*p*‐value	*R* ^2^
Secondary outcomes			
Any medical complication within 90 days	1.0 (1.0–1.0)	0.5	12.4%
Mortality within 90 days	1.0 (1.0 –1.0)	0.1	30.2%
Length of stay	1.0 (1.0–1.0)	0.6	12.5%

*Note*: Mean surgical unit volume found to be linear with outcome and modelled as continuous data.

## DISCUSSION

Larger surgical volume units experience lower re‐revision rates within 2 years than smaller units in first‐time RevKR for infection. This association appears to be independent of known confounding factors such as age, surgeon volume, sex, ASA grade, year of surgery and operation funding. This is one of the largest registry‐based studies analysing the relationship between surgical unit volume and patient‐relevant outcomes in RevKR. We are also the first study using a longitudinal data set to examine this relationship in the more complex scenario of an infected knee replacement.

Comparing previous literature to this study is complicated by the heterogeneity of the selected study population, hospital volume categories and follow‐up duration. Using data from the German national joint register, Halder et al. [13] showed the risk of re‐revision for aseptic RevKR in hospitals performing less than or equal to 12/year was 14.4% more than hospitals performing more than or equal to 53/year. Our data set using the same volume categories outlined by Halder et al. [13] showed a similar association between low hospital volume and risk of revision in our population. We also presented a similar wide variation in revision risk between the two extreme volume categories, and this provides further evidence of non‐linear relationships which must be accounted for in any volume–outcome analysis as we have presented. The reason for non‐linearity may be case mix dependent particularly at the higher volume centres, where we would expect to accept referrals of more complex cases [32]. Therefore, the true effect size may be higher than we report and requires further investigation. It is possible that a stronger correlation with volume can be established if these differences are adjusted for using additional markers for surgical and patient complexity [2]. Changes to the fields on Minimum Data Set K2 forms may see the application of complexity scores to adjust for this confounding.

Currently, the minimum volume thresholds assigned to surgical units for RevKR procedures in England and Wales are 30/year with individual surgeons needing to achieve at least 15/year [34]. Our results do not allow us to draw statistically robust estimates for these thresholds due to the continued downward trend in re‐revision rate with increasing annual unit volume. However, our results suggest first‐time revision for infection should be done by the surgical units with the most experience. As such, commissioners and subspecialty clinical leads should use this information within the practical constraints of the NHS to inform minimum volume thresholds.

There are several factors which cannot be measured that are likely to be related or contribute to the volume–outcome relationship. These are factors such as pre‐existing multidisciplinary team (MDT) working and standardisation of services, which may be utilised in larger volume units where there is a requirement for greater organisational and procedural standardisation [6]. The positive effect of MDT working on outcomes following RevKR was observed by the East Midlands Specialist Orthopaedic Network [4]. This regional collaborative approach, which was set up in 2015, provided an opportunity for surgeons working in five hospitals to identify and discuss complex cases. The decision to refer to the high‐volume regional centre was based on the clinical opinion of the referring surgeon, rather than any pre‐defined volume threshold. They found such an intervention led to a significant improvement in the re‐revision rate when compared to the rest of England [5]. Anecdotal evidence suggests other regions have been implementing a similar working approach, and it is difficult to adjust for this confounder when investigating the true volume–outcome relationship. Revision knee networks aim to combine the setting of minimum volume thresholds, procedure standardisation and collaborative practice. Registry‐based studies such as this will be well placed to observe re‐revision TKR rates in the context of this intervention.

We demonstrated no association between surgical unit caseload and mortality, serious medical complications within 90 days and hospital length of stay.

## LIMITATIONS

This study uses data from secondary data sources and as such has limitations common to observational research. Such data sources may lack granularity and accuracy. Despite this, the NJR is a mature registry and has been shown to have excellent data quality [29]. However, patients not consenting to the NJR audit were omitted from this study, potentially introducing a selection bias. All publicly funded hospital admissions, both in NHS and independent hospitals, are recorded in HES. Financial incentives exist to improve the coding accuracy within national administrative data sets such as HES [14]. Linked HES and national mortality statistics have been shown to provide accurate and clinically useful predictions for mortality up to 1 year [1].

The relationship between volume and outcome, particularly within complex joint arthroplasty, is challenging to determine, particularly when using observational data repositories such as NJR and HES. This is because we have used a broad definition of a RevKR, and the technical difficulty of procedures, which fall under this definition, is variable and is currently not recorded with adequate granularity; for example, the surgery can range from a simple exchange of components to a complex reconstruction in the presence of infection and severe bone loss. The data set in this study was minimised to include only first‐time single‐stage revisions for infection in an effort to control for this. To identify the primary diagnosis, a hierarchical classification was used to define the primary reason for revision, and this may oversimplify cases with multiple contributing factors. There are technical details of the operation which contribute to operative complexity which are not routinely collected at present. It is a limitation of the current study that it remains highly feasible that the surgical complexity can influence both primary and secondary outcomes and presently we are unable to adjust for this within our data set. Our final cohort of patients who met eligibility was comparatively small in comparison to the full first‐time revision repository.

Our exposure variable was calculated from all RevKR procedures and was not limited to our final patient cohort. We acknowledge this could introduce potential inaccuracies as they were excluded from outcome analysis. However, we believe the inclusion of these cases in caseload calculations is more representative of RevKR surgical experience particularly when overall RevKR now inform specific minimal volume targets for units and surgeons in England [15]. We acknowledge the surgical caseload calculation does not accommodate changes in unit volume between seasons or define changes in staff turnover. These factors are not addressed in the scope of this work and remain a limitation of the work. We did not control for perioperative factors at the discretion of the surgical unit (such as implant selection or choice of VTE prophylaxis). This is because surgical caseload does not in itself cause a better or worse clinical outcome, but instead may be the expression of other predictors—for example, surgical skill or perioperative decision‐making. Patient‐relevant characteristics such as body mass index and index of multiple deprivation were not accounted for in our modelling due to a large proportion of missing or invalid data (~33%).

During the modelling process, some continuous variables were modelled reflecting linear relationships with the outcome of interest despite showing initial evidence of non‐linearity. We acknowledge this may oversimplify its effect on outcome. However, subsequent testing of these variables did not provide robust evidence to determine a non‐linear relationship. The data set used in this study is historical, and this may affect the generalisability of the results as we preclude more recent up‐to‐date data. However, this was intentional to avoid the substantial impact on revision volumes due to the pandemic seen in 2020 [26]. Furthermore, revision networks were introduced in England after April 2022. These were introduced at varying time points and doses in different regions of England, which would be difficult to adjust for in our analyses.

Our primary and secondary outcomes are focused within a short timeframe. This could potentially exclude important late outcomes with reference to re‐revision rates. However, we believe the analysis of late outcomes introduces bias in the assessment of volume‐outcome relationships. Late re‐revisions may be due to expected wear of the implant or infection from other body sites. Early re‐revisions are more representative of the quality of the surgical provision. The biggest data omission in this study is the absence of PROMs. We recorded a very high record attrition with only 6% of our population recording complete pre‐ and post‐operative PROMs. This is a cause for concern, and we would encourage a review of more current NJR data to ensure this pattern has not continued.

Despite this being an observational study, a randomised controlled trial is unlikely to be feasible for various reasons, most notably the likelihood that patients would be reluctant to consent to surgery by a less experienced unit where surgery by an experienced centre was an option. Nevertheless, ‘real‐world’ data comparing regions where minimum volume standards are in place (or where regional networks triage care) and regions where this has not occurred would be invaluable in informing the debate.

## CONCLUSION

We provide evidence that a surgical unit volume–outcome relationship exists for first‐time single‐stage RevKR for infection. A system which focuses on combining the setting of minimum annual volume thresholds in addition to standard operating procedures and collaborative practice may lead to improved patient‐relevant outcomes. Further work is needed to map the complexity of revision knee practice and its relationship with minimum surgical unit volume thresholds.

## AUTHOR CONTRIBUTIONS

All authors had full access to all the data in the study and had final responsibility for the decision to submit for publication. **Alexander H. Matthews**: Conceptualisation; methodology; funding acquisition; software; project administration; investigation; data curation; formal analysis; validation; visualisation; writing—original draft; writing—review and editing. **William K. Gray**: Conceptualisation; methodology; investigation; validation; supervision; writing – review and editing. **Jonathan P. Evans**: Conceptualisation; supervision; writing—review and editing. **Ruth Knight**: Data curation; methodology. **Jonathan T. Evans**: Supervision; writing—review and editing. **Sarah E. Lamb**: Supervision; writing—review and editing. **Tim Briggs**: Funding acquisition; supervision; writing—review and editing. **Andrew Porteous**: Funding acquisition; writing—review and editing. **Shiraz A. Sabah**: Conceptualisation; data curation; validation; writing—review and editing; supervision. **Abtin Alvand**: Supervision; writing—review and editing. **Andrew Price**: Conceptualisation; funding acquisition; supervision; writing—review and editing. **Andrew D. Toms**: Conceptualisation; funding acquisition; supervision; writing—review and editing.

## CONFLICT OF INTEREST STATEMENT

The authors declare no conflicts of interest.

## ETHICS STATEMENT

Ethical approval was obtained from the London‐Bromley Research Ethics Committee (20/LO/0428). Data access approvals were obtained from the National Joint Registry for England, Wales, Northern Ireland, the Isle of Man and the States of Guernsey (NJR) (RSC2017/26). Patients who did not consent to the NJR audit were not included.

## Data Availability

The data sets generated and analysed in the current study are not publicly available due to data protection regulations. Access to data is limited to the researchers who have obtained permission for data processing. Further inquiries can be made to the National Joint Registry research team.
